# Seasonal Patterns of Japanese Encephalitis and Associated Meteorological Factors in Taiwan

**DOI:** 10.3390/ijerph14111317

**Published:** 2017-10-29

**Authors:** Che-Liang Lin, Hsiao-Ling Chang, Chuan-Yao Lin, Kow-Tong Chen

**Affiliations:** 1 Internal Medicine Chest Division, Chi-Mei Medical Center, Liouying, Tainan 736, Taiwan; lilytang19@gmail.com; 2 Division of Infection Control and Biosafety, Centers for Disease Control, Ministry of Health and Welfare, Taipei 104, Taiwan; hlchang@cdc.gov.tw; 3 School of Public Health, National Defense Medical Center, National Defense University, Taipei 117, Taiwan; 4 Research Center for Environmental Changes, Academia Sinica, 115, Taiwan; yao435@gate.sinica.edu.tw; 5 Department of Occupational Medicine, Tainan Municipal Hospital (Managed by Show Chwan Medical Care Corporation), Tainan 701, Taiwan; 6 Department of Public Health, College of Medicine, National Cheng Kung University, Tainan 701, Taiwan

**Keywords:** infectious diseases, climate, modeling, mosquito

## Abstract

The persistent transmission of Japanese encephalitis virus (JEV) in Taiwan necessitates exploring the risk factors of occurrence of Japanese encephalitis (JE). The purpose of this study was to assess the relationship between meteorological factors and the incidence of JE in Taiwan. We collected data for cases of JE reported to the Taiwan Centers for Disease Control (Taiwan CDC) from 2000 to 2014. Meteorological data were obtained from the Taiwan Central Weather Bureau. The relationships between weather variability and the incidence of JE in Taiwan were determined via Poisson regression analysis and a case-crossover methodology. During the 15-year study period, a total of 379 cases of JE were reported. The incidence of JE showed significant seasonality, with the majority of cases occurring in summertime (for oscillation, *p* < 0.001). The number of JE cases started to increase at temperatures of 22 °C (r^2^ = 0.88, *p* < 0.001). Similarly, the number of JE cases began to increase at a relative humidity of 70–74% (r^2^ = 0.75, *p* < 0.005). The number of JE cases was positively associated with mean temperature and relative humidity in the period preceding the infection. In conclusion, the occurrence of JE is significantly associated with increasing temperature and relative humidity in Taiwan. Therefore, these factors could be regarded as warning signals indicating the need to implement preventive measures.

## 1. Introduction

Japanese encephalitis (JE) is caused by the Japanese encephalitis virus (JEV). Japanese encephalitis virus, belonging to the *Flaviviridae* family, is transmitted by mosquitoes from animals to humans [1]. The main transmission vectors of the JEV are *Culex* mosquitoes, particularly *Culex tritaeniorhynchus* (*Cx. tritaeniorhynchus*), and the main vertebrate hosts for amplifying JEV are pigs and ardeid birds [2,3]. The illness spectrum of JE in humans ranges from asymptomatic infection to a devastating encephalitis syndrome that is associated with appreciable mortality and frequent central nervous system (CNS) sequelae in survivors [4].

JE is an important cause of viral encephalitis in most Asian countries, particularly in South Asia, Southeast Asia, and East Asia [5,6]. Several countries in these regions have significantly reduced the morbidity of JE through several intervention measures, including early diagnosis, prompt treatment, national immunization, and effective vector control [7,8]. However, more than 3 billion people still live in JE-endemic countries, and an estimated 67,900 cases occur annually [5]. Approximately 20–30% of JE cases are fatal and 30–50% of survivors have significant neurologic sequelae [6,9].

In Taiwan, a comprehensive vaccination campaign against JE was launched in the 1960s, during which all children aged less than 3 years old received two doses of the JE vaccine [10,11]. After 1974, the number of vaccination doses was increased to three, with a booster dose administered one year after the two primary doses. After 1976, a fourth booster dose was administered to children during their first year at elementary school. After 1980, the mainly children older than 15 months was target population for JE vaccinations. The vaccination was administered in two doses separated by an interval of 2 weeks, which were administered between March and May, before the epidemic season, and were followed by a booster dose one year later, with a final booster dose (the fourth dose) when the child entered the elementary school. Before 1987, an inactivated vaccine derived from a Nakayama-NIH strain JEV-infected mouse brain was the only vaccine used. A version derived from an inactivated freeze-dried Beijing strain was introduced in 1988, but the Nakayama strain of the vaccine has remained the dominant vaccine on the market. Despite the current vaccination program, cases of JE still occur in Taiwan [10].

The occurrence of vector-borne diseases (VBD) is strongly driven by environmental factors [8,12,13]. Among the most relevant environmental factors are climate, land use and land cover [14]. Several recent studies have shown an increasing trend of the epidemic potential and length of the transmission season of VBDs in temperate regions and tropical highlands under different climate change scenarios [15,16,17]. Although these phenomena have not been adequately explained, the seasonality of JE suggests that meteorological factors might play a key role in its occurrence [10,14,18].

Moreover, the Intergovernmental Panel on Climate Change (IPCC) indicates that local changes in temperature and rainfall will continue to alter the distribution of disease vectors and the risk of VBDs [16,17]. However, heterogeneity in VBD transmission is observed at every spatial scale, ranging from small islands to continents. This heterogeneity is determined by the ecology and biogeography of the vectors, soil types, urbanization, local adaptation to temperature, and host communities [17,19]. The effects of meteorological factors on infectious diseases have attracted global attention in the context of climate change in recent years.

A further understanding of the relationships between meteorological factors and the occurrence of JE could help improve both disease forecasting and preventive efforts. However, few studies have investigated the effects of meteorological factors on the occurrence of JE in Taiwan. The purpose of this study was to assess the relationships between weather-related factors and the number of JE cases in Taiwan.

## 2. Methods

### 2.1. Study Area

Taiwan is an island in East Asia and is located between 21°45’ N and 25°56’ N. The Tropic of Cancer (23.5° N) runs straight through Chiayi City, which is situated in south central Taiwan and divides the entire island into two climate zones. Taiwan includes a total land area of 35,980 km^2^ and approximately 2.3 million people, giving an average population density of 635 individuals per km^2^. The northern part of Taiwan belongs to the sub-tropical climate zone, whereas the southern part belongs to the tropical climate zone. Consequently, the weather in Taiwan is relatively warm, and high humidity occurs throughout the year [20].

This study was approved by the Institutional Review Board of the Show Chwan Memorial Hospital, Changhua, Taiwan (SCMH_IRB No. 1040903).

### 2.2. Surveillance for Japanese Encephalitis Infection

The data used in this study have been previously published by Chang et al. [10]. Briefly, JE has been categorized as a notifiable infectious disease since 1955. Physicians are required to report all cases that meet the case definition of JE, collect samples, and send them to the Centers for Disease Control of Taiwan (Taiwan CDC) within one week of the case being reported for examination [11].

We collected data from all JE-confirmed cases reported to the Taiwan CDC from January 2000 to December 2014. The reported information included patient age, sex, area of residence, geographic location of exposure, travel history, vaccination status, and date of JE onset.

### 2.3. Case Definitions

A clinical case was defined as a person of any age with an acute onset of fever and a change in mental status and/or a new onset of seizures (excluding simple febrile seizures) at any time of the year [6,10].

A confirmed case was defined as a clinical case with a positive laboratory test (presence of IgM antibodies specific to the JE virus in a single sample of CSF or serum; and/or a four-fold increase in IgG antibodies; and/or the detection of JE virus antigens in tissue via immunohistochemistry; or the detection of the JE virus genome in serum, plasma, blood, CSF or tissue samples; or that met the clinical case definition and was epidemiologically linked to a confirmed case [10,11,21].

### 2.4. Meteorological Data

Complete meteorological data, including the maximum and minimum daily mean temperatures, relative humidity, vapor pressure, precipitation, and daylight hours, between January 2000 and December 2014 were obtained from the Taiwan Central Weather Bureau (http://www.cwb.gov.tw). We used the regional meteorological data value for each calendar week obtained from all 17 weather stations across the island, excluding stations in isolated islands and areas in mountains, linked to each case depending on their regions of residence.

### 2.5. Statistical Analysis

We calculated the annual incidence of JE by dividing the number of reported JE cases by the mid-year population of individuals of the same age, as reported between 2000 and 2014 in Taiwan census data. This was expressed as the number of JE cases per 1,000,000 individuals. Seasonal trends in the occurrence of JE were assessed using Poisson regression models that incorporated terms for the calendar year, as well as sine and cosine to assess time trends and seasonality of JE using the monthly aggregate case number as the response variable. In addition to the time trends and seasonality, we added the temperature, relative humidity, vapor pressure, precipitation, and daylight hours into the models [20,22], such that:

(1) $$E\left[Y_{i}\left(t\right)\right]=\exp\{\begin{array}{c} \alpha +\beta_{1}\times year_{i}\left(t\right)+\beta_{2}\left[\sin\left(2\pi \times \left(t\right)/12\right)\right]+\beta_{3}\left[\cos\left(2\pi \times \left(t\right)/12\right)\right]+\beta_{4}\left[temperature_{i}\left(t\right)\right]\\ +\beta_{5}\left[vapor\;pressure_{i}\left(t\right)\right]+\beta_{6}\left[humidity\left(t\right)\right]+\beta_{7}\left[precipitation\left(t\right)\right]+\beta_{8}\left[sunshine\;hours\left(t\right)\right] \end{array}\}$$

(2) $$Y_{i}\left(t\right)=\{\begin{array}{l} =1\;\mathrm{if}\;\mathrm{year}=i\\ =0\;\mathrm{otherwise} \end{array}\;\mathrm{Month}\left(t\right)=1\;\mathrm{if}\;t=1,\;\mathrm{so}\;\mathrm{month}\;\left(t\right)=t$$

in which *E*[*Y_i_*(*t*)] denotes the expected case counts at month *t* in year *i*. α is a constant value, each *β* term denotes a regression coefficient for a year or month, *t* indicates the months between January 2000 and December 2014, and *i* indicates the 15 years during the years 2000 and 2014. The function year*_i_*(*t*) denotes whether it is *year_i_* (1 = yes, 0 = no). The function month (*t*) indicates a month number (i.e., 1 to 12 for January to December). Temperature*_i_*(*t*) is the temperature at month *t* in year *i*; likewise, relative humidity*_i_*(*t*) is the relative humidity at month *t* in *year_i_*. We used the construction of univariable and multivariable Poisson regression models to evaluate the correlation between monthly number of JE cases and weather exposure. We also used oscillatory seasonal smoothers for smoothing to account for annual variations during the 15-year study period. We used Akaike’s information criterion (AIC) to optimize the knots within the spline model to avoid the pitfalls associated with both overfitting and underfitting [23]. A backwards-elimination algorithm was conducted in multivariable models, in which covariates were retained at *p* ≤ 0.20 [23].

To investigate the relationship between JEV infections and various temperatures and relative humidity levels, we estimated the incidence of JE at various temperatures and relative humidity levels. According to previous studies [18,24,25], we assumed that the survival and transmission of the JE virus would change as temperature and relative humidity changed, and this might therefore affect the infectivity of the JE virus in a defined population. The average incidence of JE (*N_T_*) in various temperature domains (T to T + ΔT) was estimated using the following formula [20,22]:

(3) $$N_{T}=\frac{\sum_{i}^{n}C_{i}f\left(t_{i}\right)}{\sum_{i}^{n}f\left(t_{i}\right)}$$

Here, *i* denotes an index from 0 to *n*, *t_i_* is the average temperature for the *i*th 7-day period, *C_i_* is the total cases of JE for the *i* + 2nd 7-day period, and *f* (*t_i_*) is a function with the following results:

(4) $$f\left(t_{i}\right)\{\begin{array}{l} =1\;\mathrm{when}\;T<t_{i}\leq Τ+\Delta T\\ =0\;\mathrm{otherwise} \end{array}$$

The numerator on the right side of the equation is the sum of all *C_i_* comprising the 7-day average temperatures (*t_i_*) within the temperature domain of T to T+ΔT during the study period. The denominator is the total number of JE cases with T < *t_i_* ≤ T + ΔT during the same study period.

Similarly, the average incidence of JE (*N_h_*) in various relative humidity domains (H to H + ΔH) was assessed using the following formula:

(5) $$N_{H}=\frac{\sum_{i}^{n}C_{i}f\left(h_{i}\right)}{\sum_{i}^{n}f\left(h_{i}\right)}$$

Here, *i* is a sequence from 0 to *n*, *h_i_* is the average relative humidity for the*i*th 7-day period, *C_i_* is the total cases of JE from the *i* + 2nd 7-day period, and *f* (*h_i_*) is a function with the following results:

$$f\left(h_{i}\right)\{\begin{array}{l} =1\;\mathrm{when}\;H<h_{i}\leq Η+\Delta H\\ =0\;\mathrm{otherwise} \end{array}$$

We used a case-crossover analysis to further investigate the acute effects of meteorological exposure on the occurrence of JE [26]. This design is characterized by self-matching, wherein cases serve as their own controls [27,28]. This specifically provides a means to evaluate the acute effects of brief exposures [26,29]. In this way, each subject’s exposure prior to a case-defining event was compared with his or her own exposure during a control period when he/she had not yet been diagnosed as a case. A case day was defined as the day on which the first symptom of JE presented [28,30], and the case period was defined as 0–14 days prior to that day. The control day was selected two to four weeks before the case date (14–29 days prior to the case day). The possible effect period was estimated according to the incubation period of JE, which is approximately 7 days (range: 5–15 days) [8]. The average daily values of the meteorological factors were used as exposures, as were aggregated or mean values of the meteorological factors.

The analysis of case-crossover data is an application of standard methods for stratified data analysis. A conditional logistic regression analysis was performed to determine exposure odds ratios (ORs) as estimates of incidence rate ratios and 95% confidence intervals (CIs) associated with meteorological variables [31].

To study the possibility of interactions (effect modification) from the demographic characteristics of patients, we developed multiplicative interaction terms and incorporated them into a logistic regression model [32]. We used SAS software Version 9.2 (SAS Institute Inc., Cary, NC, USA) to perform all statistical analyses. A *p*-value of less than 0.05 was considered statistically significant.

## 3. Results

### 3.1. Epidemiological Characteristics of Patients with Japanese Encephalitis

Table 1 shows the number and annual incidence rate of JE cases by gender, age, region, and season. Between January 2000 and December 2014, a total of 379 patients with JE were diagnosed by physicians in Taiwan. All cases were indigenous. The incidence rate (cases per 1,000,000 individuals per year) of JE was 1.105 (range: 0.59 to 1.61). JEV predominantly affected men compared to women (1.366 vs. 0.839). The incidence of JE increased as patient age increased, with a peak (1.491) in the age range of 20–59 years. The highest and lowest incidences of JE were 4.829 and 0.492 in the eastern and the northern region, respectively, in Taiwan. The highest incidence of JE was 0.902 in the summer (June to August), and followed by 0.114 in the spring, and 0.090 in the autumn.

### 3.2. Seasonality and Effects of Weather Factors

After oscillatory or cubic spline smoothers were incorporated into the model, mean temperature and mean relative humidity were the only factors found to be independently associated with JE infection.

**Figure 1:**
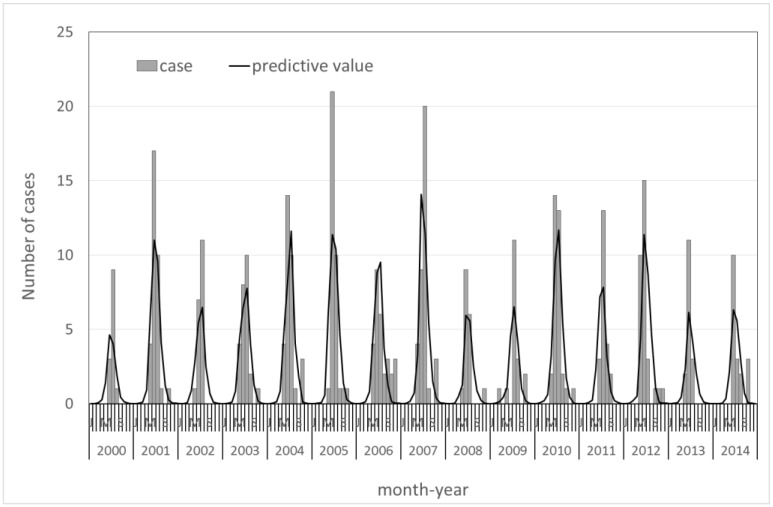
Trends in monthly Japanese encephalitis cases from 2000–2014 in Taiwan.

Figure 1 shows the actual and predicted monthly reported cases of JE. There was a seasonal pattern of JE infection (for seasonal oscillation, *p* < 0.001), but no distinct annual trends in this model. In general, the overall trends of the predicted monthly reported case numbers fit the actual trends well (Pearson chi-squared = 50.78; *p* > 0.05).

The association between meteorological factors and the incidence of JE is presented in Table 2. Univariate analysis using Poisson models showed several meteorological factors associated with the incidence of JE; however, after annual trends and oscillatory seasonal smoothers were incorporated into the models, only the mean temperature and relative humidity were independently associated with the incidence of JE (Table 2). Age and sex did not modify temperature or relative humidity effects.

### 3.3. Temperature, Relative Humidity, and Occurrence of JE

**Figure 2:**
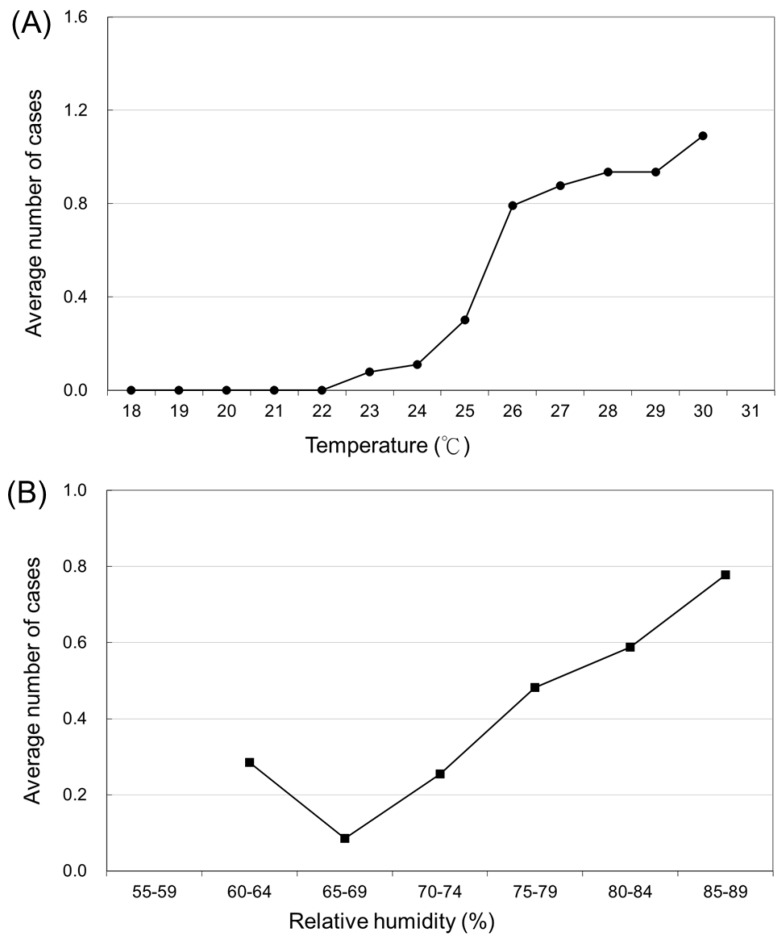
(**A**) The average number of Japanese encephalitis virus infections in various temperature domains. (**B**) The average number of Japanese encephalitis virus infections in various relative humidity domains.

The relationship between occurrence (case count) of JE and temperature is shown in Figure 2A. The occurrence of JE changes with different temperatures. The number of JE cases began to rise at a temperature of 22 °C (r^2^ = 0.88, *p* < 0.001). The average case count (*N_T_*) increased by 14.4% (95% CI: 7.4–21.4%) for each 1 °C increase in temperature.

Figure 2B presents the association between the variation in the number of JE cases and the various relative humidity domains (*N_h_*). The number of JE cases began to increase at a relative humidity of 70–74% (r^2^ = 0.75, *p* < 0.05). An increase in relative humidity of 5% was correlated with a 9.8% increase in JE cases (95% CI: 1.0–18.6%).

### 3.4. Acute Meteorological Effects Using Case-Crossover Analysis

**Figure 3:**
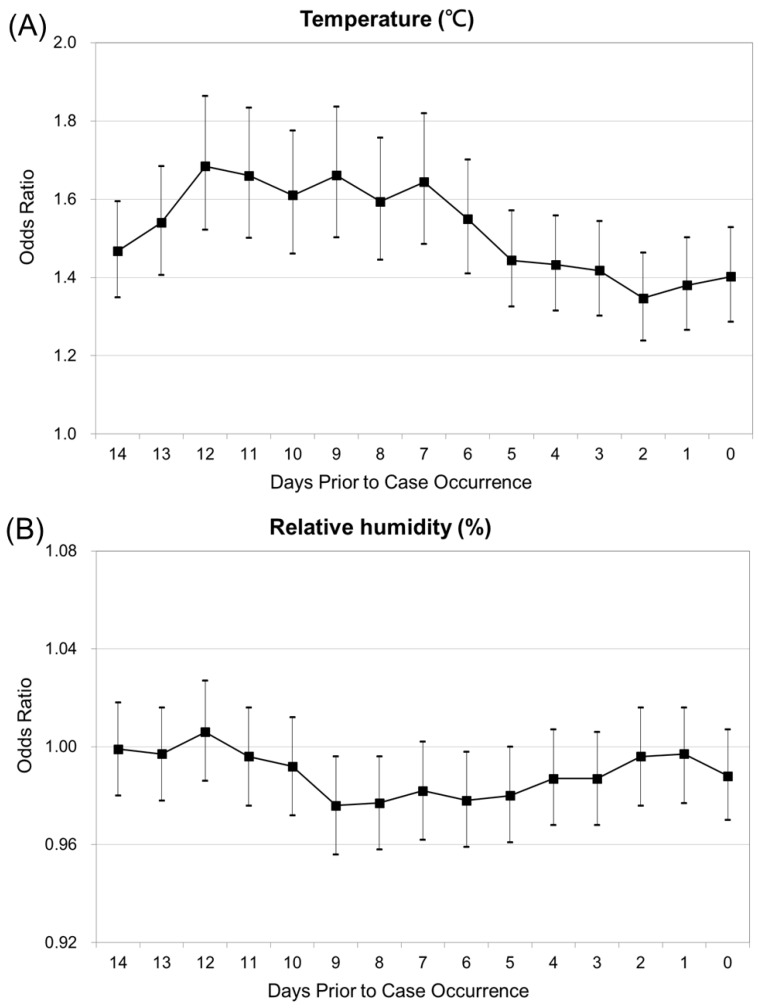
(**A**) Conditional logistic regression analysis for Japanese encephalitis, with temperature as explanatory variables. (**B**) Conditional logistic regression analysis for Japanese encephalitis, with relative humidity explanatory variables.

Figure 3A presents the relationship between the risk of JE virus infection and per degree increase in temperature from days 3–7, plateau till 12 days, and then decline. Figure 3B shows the risk of JE infection to steadily drop till day 9, and increase till day 12, and then decline, as per unit (5%) increase in relative humidity. The increased occurrence of JE was significantly associated with increased mean temperature and relative humidity.

## 4. Discussion

The epidemiological evidence demonstrated that JE remains an important public health problem in Taiwan [5,10]. We analyzed JE data reported to the Taiwan CDC from 2000 to 2014 using Poisson regression analysis and a case-crossover methodology. We found that the incidence of JE was highest during the summer months, but that the incidence was positively correlated with increased mean temperature and mean relative humidity in both long-term and acute effect analyses. In this study, we also identified the importance of meteorological exposure in determining the incidence and seasonality of JE in developed countries, such as Taiwan.

JEV is an arthropod-borne virus (arbovirus) that is transmitted in an enzootic cycle among mosquito vectors and vertebrate hosts [33]. The emergence of JE is driven by environmental factors, such as climate [8]. Various patterns of seasonality in the occurrence of JE have been reported in various countries. In temperate countries (e.g., China, Japan, Nepal, and Korea), seasonal JE outbreaks occur in conjunction with the temperature and rainfall increase in the summer months, when JEV is detectable in mosquitoes, pigs, and birds. On the other hand, in tropical and subtropical countries (e.g., Indonesia, Malaysia, the Philippines, and Vietnam), sporadic JE cases occur year-round, with a peak during the rainy season [8,14]. Our results showed a distinct seasonal pattern of JEV infection, with a peak occurrence in the summer (June, July, August) months. This result was similar to Southern China [18,34], India [15] and Nepal [24].

In this study, the occurrence of JEV infection began to increase at 22 °C. We also found that the number of JE cases started to increase at a relative humidity of 70–74%, with transmission assumed to be due to biting by an infected mosquito. It has been reported that the activity of the JEV is affected by the temperature and relative humidity [8,33]. The mechanism underlying the effects of temperature and relative humidity in the transmission of JEV remains unclear.

In our study, male patients had a higher annual incidence rate of JE than female patients (1.37 vs. 0.84 per 1,000,000). Hsu’s study [35] showed the odds of having JE neutralizing antibodies are higher in men than in women (ratio = 1.25, 95% CI: 1.12–1.40) in Taiwanese people. However, no sex differences were reported in the incidence of JE cases in India [13,21]. We are unable to definitively explain this epidemiological finding in Taiwan, but postulate that contributing factors may include different behavioral patterns or different reporting patterns.

Children less than 15 years of age were considered a high-risk group for JE in South, East, Southeast Asia and Australasia because most adults are immune [5]. Since childhood JE vaccination programs have not been fully implemented across the geographic range of JEV, the target population for infections has shifted from children to adults in several countries, in contrast to the pre-vaccination era [36,37]. In our study, the highest incidence rate of JE occurred in the age range of 20–59 years old.

In our study, a high percentage (42.7%) of JE cases occurred in the southern region of Taiwan. There are several possible reasons for this, which are discussed below: (1) In Taiwan, most confirmed cases of JE involved patients who lived near paddy fields or pig farms [38], and southern Taiwan is an agricultural region. Compared with other regions, there are more pig farms near rice paddy fields as well as wetland habitats for water birds in southern Taiwan. These provide suitable environments for maintaining the JEV infection cycle [39,40]. (2) In Taiwan, almost all dengue fever cases (approximately >95%) over the last decade occurred in the southern region of Taiwan [11]. This fact, along with the high proportion of JE cases, suggests that mosquitoes and the JE virus are well-adapted to this region. (3) Finally, the higher proportion of cases in the southern region of Taiwan is also a reflection of the population numbers.

This study has several limitations. First, the public health surveillance data may be incomplete. It is believed that many notifiable infectious diseases (e.g., JE) are underreported [41]. A reporting bias may occur anywhere in the reporting chain. This bias would occur if weather effects were somehow correlated with the likelihood of disease reporting [23]. Since doctors may be more likely to test for JE when they consider it high season for it, and thus mild cases in the beginning of the season are more likely to be missed. Second, it was difficult to obtain weather data in all counties in Taiwan. After excluding stations in isolated islands and areas in the mountains, only 17 weather stations were ultimately included in our analysis. These weather data may not represent the true status of weather exposure in the individual areas in Taiwan. The results supported the null hypothesis due to non-differential misclassification. The effects of meteorological exposure on the occurrence of JE in this study were most likely underestimated [27].

In summary, the seasonal pattern of JEV infection in Taiwan was confirmed, and meteorological factors that might contribute to the observed seasonality, including the mean temperature and relative humidity, were evaluated. Public health authorities could regard the results of the threshold estimation as a warning signal, and by applying these results with a prediction model for long-term trends, they could develop and deploy public health interventions before early summer to reduce the risk of infection and spread of the JE virus. JE has attracted growing attention because several unexpected outbreaks occurred in many countries in the 2000s [15,24]. Our findings demonstrate the importance of meteorological factors in determining JE case occurrence and can help explain the notable seasonal pattern of JE.

## 5. Conclusions

The occurrence of JE is significantly associated with increasing temperature and relative humidity in Taiwan. Therefore, these factors could be regarded as alert signals indicating the need to implement preventive strategies.

## Figures and Tables

**Table 1:** Demographic characteristics of patients with Japanese encephalitis in Taiwan, 2000–2014.

Variables	Case Number (%) *N* = 379	Annual Incidence Rate Per 1,000,000
Total	379	1.105
Gender		
Male	237 (62.5)	1.366
Female	142 (37.5)	0.839
Age groups (years)		
≤9	7 (1.8)	0.183
10–19	13 (3.4)	0.270
20–59	311 (82.1)	1.491
≥60	48 (12.7)	0.975
Region of residence		
Northern	85 (22.4)	0.492
Central	90 (23.7)	1.367
Southern	162 (42.7)	1.505
Eastern	42 (11.1)	4.829
Seasons		
Summer	309 (81.5)	0.902
Spring	39 (10.3)	0.114
Autumn	31 (8.2)	0.090
Winter	0 (0.0)	0.000

**Table 2:** Weekly weather patterns 8–14 days prior to symptom onset and the incidence of JE virus infection in Taiwan, 2000–2014.

Meteorological Element	Univariable Models			Multivariable Model Including Oscillatory Seasonal Smoothers and Annual Trend		
	IRR	95% CI	*p* Value	IRR	95% CI	*p* Value
Mean temperature, 1 °C	1.43	1.37, 1.50	<0.001	1.30	1.06, 1.60	0.013
Mean difference in temperature, 1 °C	0.99	0.82, 1.20	0.945			
Mean relative humidity, 5%	1.23	1.18, 1.28	<0.001	1.10	1.04, 1.18	0.002
Mean vapor pressure, 1 hPa	1.30	1.26, 1.34	<0.001			
Mean cumulative precipitation, 1 mm	1.11	1.10, 1.13	<0.001			
Mean precipitation time, 1hr	0.95	0.86, 1.05	0.323			
Mean cumulative daylight hours, 1 h	1.46	1.37, 1.56	<0.001			
Mean sunshine rate, 1%	1.03	1.02, 1.04	<0.001			

Note: JE: Japanese encephalitis; IRR: incidence rate ratio; CI: confidence interval.
